# THE PROMISE AND PERIL OF RUNNING A CENTER/PROGRAM ON AGING

**DOI:** 10.1093/geroni/igae098.0837

**Published:** 2024-12-31

**Authors:** Pamela Teaster, Pamela Saunders, Pamela Saunders

**Affiliations:** Chair; Co-Chair; Discussant

## Abstract

Gerontology centers abound across the country in various forms. Despite an unprecedented rise in the worldwide population of older adults, some programs flourish while others struggle. This symposium examines the promise of running gerontology programs as well as the perils of doing so. Offering examples and insights, Dr. Pamela Teaster (Virginia Tech) will discuss how a nearly 50 year old gerontology center has morphed since its beginnings to planning for its 50th anniversary celebration. Dr. David Burdick (Stockton University) will present the long view of leading a gerontology program for 25 years and a center for 15 years, and along with Dr. Christine Ferri will discuss the experience of succession planning for both program and center. Dr. Christopher Kelley (University of Nebraska at Omaha) describes the importance of partnerships with public and private stakeholders at the national, state, and local levels. Dr. Pamela Saunders (Georgetown University) will make observations about differences and common themes among the presentations.

